# Steric hindrance in the upper 50 kDa domain of the motor Myo2p leads to cytokinesis defects in fission yeast

**DOI:** 10.1242/jcs.205625

**Published:** 2018-01-01

**Authors:** Saravanan Palani, Ramanujam Srinivasan, Paola Zambon, Anton Kamnev, Pananghat Gayathri, Mohan K. Balasubramanian

**Affiliations:** 1 Centre for Mechanochemical Cell Biology and Division of Biomedical Sciences, Warwick Medical School, University of Warwick, Coventry CV4 7AL, UK; 2 School of Biological Sciences, National Institute of Science Engineering and Research (NISER), Bhubaneswar, Odisha 752050, India; 3 Biology Division, Indian Institute of Science Education and Research (IISER), Pune 411 008, India

**Keywords:** Actomyosin ring, Cytokinesis, Fission yeast, Myosin II

## Abstract

Cytokinesis in many eukaryotes requires a contractile actomyosin ring that is placed at the division site. In fission yeast, which is an attractive organism for the study of cytokinesis, actomyosin ring assembly and contraction requires the myosin II heavy chain Myo2p. Although *myo2*-E1, a temperature-sensitive mutant defective in the upper 50 kDa domain of Myo2p, has been studied extensively, the molecular basis of the cytokinesis defect is not understood. Here, we isolate *myo2*-E1-Sup2, an intragenic suppressor that contains the original mutation in *myo2*-E1 (G345R) and a second mutation in the upper 50 kDa domain (Y297C). Unlike *myo2*-E1-Sup1, a previously characterized *myo2*-E1 suppressor, *myo2*-E1-Sup2 reverses actomyosin ring contraction defects *in vitro* and *in vivo*. Structural analysis of available myosin motor domain conformations suggests that a steric clash in *myo2*-E1, which is caused by the replacement of a glycine with a bulky arginine, is relieved in *myo2*-E1-Sup2 by mutation of a tyrosine to a smaller cysteine. Our work provides insight into the function of the upper 50 kDa domain of Myo2p, informs a molecular basis for the cytokinesis defect in *myo2*-E1, and may be relevant to the understanding of certain cardiomyopathies.

## Abstract (precis)

**Summary:** An unbiased genetic screen uncovers a mutation that reverses a steric clash in the myosin II motor head, which was previously reported to cause defective actomyosin ring-dependent cytokinesis in fission yeast.

## INTRODUCTION

Eukaryotic cytokinesis is achieved using an actomyosin-based ring whose contraction generates tension to divide one cell into two (Balasubramanian et al., 2004; Cheffings et al., 2016; Laporte et al., 2010; Pollard and Wu, 2010). F-actin, myosin II and a large number of actin and myosin binding proteins and modulators are components of the actomyosin ring (Bezanilla et al., 1997; Cheffings et al., 2016; Kitayama et al., 1997; May et al., 1997; Motegi et al., 1997; Wu and Pollard, 2005; Wu et al., 2006). Of these, myosin II has gained considerable attention owing to its function as a motor that generates tension (Bezanilla et al., 1997; De Lozanne and Spudich, 1987; Herman and Pollard, 1979; Kitayama et al., 1997; Mabuchi and Okuno, 1977; Motegi et al., 1997, 2000; Naqvi et al., 2000). Consistently, mutants affected in myosin II heavy and light chains affect cytokinesis (Balasubramanian et al., 1998; Bezanilla et al., 1997; Bi et al., 1998; Karess et al., 1991; Kitayama et al., 1997; Knecht and Loomis, 1987; Lippincott and Li, 1998; McCollum et al., 1995; Motegi et al., 1997; Naqvi et al., 2000; Palani et al., 2017; Zambon et al., 2017). Myosin II function in cytokinesis has parallels with its function in cardiac smooth muscle, and defects in myosin II can cause hypertrophic cardiomyopathy and dilated cardiomyopathy (Huang and Szczesna-Cordary, 2015; Kim et al., 2005; Ma et al., 2012; Tang et al., 2016). Thus, understanding myosin II structure and function can provide insight into cytokinesis and further afield.

The fission yeast *Schizosaccharomyces pombe* is an attractive model organism to understand eukaryotic cytokinesis mechanisms because it divides using a contractile actomyosin ring (Goyal et al., 2011; Pollard, 2008; Vavylonis et al., 2008). In *S. pombe*, cytokinesis involves two myosin II heavy chains, Myo2p and Myp2p (Bezanilla et al., 1997, 2000; Kitayama et al., 1997; Motegi et al., 1997). Of these Myo2p is essential for cell viability, actomyosin ring assembly and contraction (Balasubramanian et al., 1998; Kitayama et al., 1997). Furthermore, Myo2p has motor activity-dependent and -independent (potentially as an actin cross-linker) functions during actomyosin ring assembly and a motor activity-dependent function during ring contraction (Palani et al., 2017). By contrast, Myp2p is non-essential and plays an ancillary role in actomyosin ring contraction at lower temperatures (Bezanilla et al., 1997; Motegi et al., 1997). Much of the *in vivo* function of Myo2p has been gleaned from the characterization of the temperature-sensitive allele, *myo2*-E1 allele (Balasubramanian et al., 1998). Molecular analysis of *myo2*-E1 has identified a single mutation, causing substitution of a glycine residue in the upper 50 kDa domain of the Myo2 head with a bulky arginine residue (Balasubramanian et al., 1998). This has led to the proposal that the arginine residue in *myo2*-E1 may sterically clash with a tyrosine in position 297 (Stark et al., 2013). However, although this view is consistent with the structural modelling, it has been neither tested nor validated via independent means.

Here, in an unbiased genetic screen for suppressors of poor growth of *myo2*-E1, we identified a mutant, *myo2*-E1-Sup2, in which tyrosine 297 (which causes steric hindrance with arginine 345 in *myo2*-E1) is replaced by a relatively smaller cysteine residue. *myo2*-E1-Sup2 supports actomyosin ring contraction *in vivo*, in the presence or absence of Myp2p, and also allows ATP dependent actomyosin ring contraction *in vitro*. These observations provide a molecular mechanism for cytokinesis defects in *myo2*-E1.

## RESULTS AND DISCUSSION

**Fig. 1.**
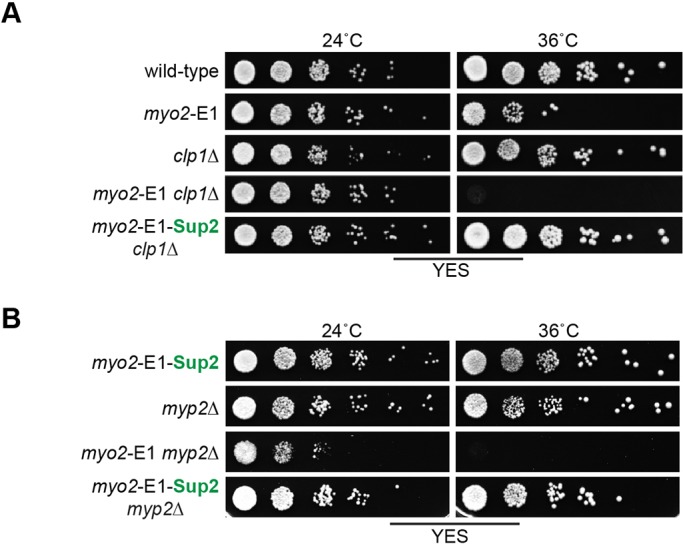
***myo2*-E1-Sup2, unlike *myo2*-E1, is viable and forms colonies at the non-permissive temperature.** (A) Serial dilutions (10 fold) of wild-type, *myo2*-E1, *clp1*Δ, *myo2*-E1 *clp1*Δ cells and the intragenic suppressor *myo2*-E1-Sup2 *clp1*Δ, spotted onto YEA plates and grown for 3 days at 24°C and 36°C. (B) Serial dilutions (10-fold) of wild-type, *myo2*-E1, *myp2*Δ, *myo2*-E1 *myp2*Δ cells and the intragenic suppressor *myo2*-E1-Sup2 *myp2*Δ, spotted onto YEA plates and grown for 3 days at 24°C and 36°C.

*myo2*-E1 grows and forms colonies at the permissive temperature of 24°C and does so slowly at the restrictive temperature of 36°C, when cells become multinucleated as a result of the assembly of improper actomyosin rings (Balasubramanian et al., 1998; Palani et al., 2017). The Clp1p phosphatase is non-essential, but its presence allows cells with weak cytokinesis defects to remain viable for long periods in a cytokinesis-competent phase, by slowing down cell division (Mishra et al., 2004). To aid identification of suppressor mutations, we used a *myo2*-E1 *clp1*Δ strain, which we have previously shown to be defective for colony formation at 36°C. Using *myo2*-E1 *clp1*Δ as a starting strain, we isolated 20 suppressors, all of which were intragenic. Of these, *myo2*-E1-Sup1 (G345R Q640H F641I) has been described previously (Palani et al., 2017). Sixteen out of the 20 suppressors contained the original G345R mutation and a second mutation that predicted replacement of tyrosine 297 with cysteine (Y297C). Fig. 1A shows the ability/inability of wild-type, *myo2*-E1, *clp1*Δ, *myo2*-E1 *clp1*Δ and *myo2*-E1-Sup2 *clp1*Δ to form colonies. Importantly, whereas *myo2*-E1 *clp1*Δ failed to form colonies at 36°C, *myo2*-E1-Sup2 *clp1*Δ was capable of doing so at 36°C (Fig. 1A).

We then generated the *myo2*-E1-Sup2 strain without the *clp1*Δ mutation to further characterize this mutant. This strain was capable of colony formation at 36°C (Fig. 1B). In previous work, we described *myo2*-E1-Sup1, whose viability at 36°C was dependent on Myp2p (Palani et al., 2017). To investigate if this growth of *myo2*-E1-Sup2 at 36°C was dependent on Myp2p, we generated a *myo2*-E1-Sup2 *myp2*Δ strain. Whereas *myo2*-E1 *myp2*Δ failed to form colonies at 36°C, *myo2*-E1-Sup2, *myp2*Δ and the double mutant *myo2*-E1-Sup2 *myp2*Δ formed colonies at 36°C (Fig. 1B). Thus, viability of *myo2*-E1-Sup2 is not dependent on Myp2p function. These experiments established that introduction of the Y297C mutation in *myo2*-E1 reversed the inability of these cells to form colonies at 36°C.

**Fig. 2.**
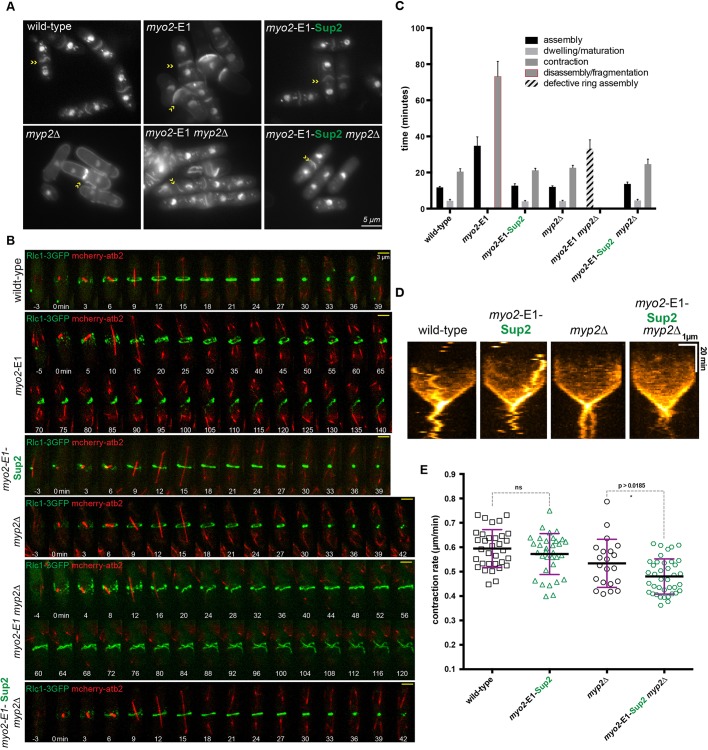
***myo2*-E1-Sup2 fully restores the actomyosin ring assembly and contraction in the presence or absence of the non-essential myosin heavy chain Myp2p.** (A) Log-phase cells were grown at 24°C and shifted for 3-4 h to 36°C before 4% paraformaldehyde (PFA) fixation. DAPI and Anillin Blue staining was used to visualize the nucleus and septum of wild-type, *myo2*-E1, *myo2*-E1-Sup2, *myp2*Δ, *myo2*-E1 *myp2*Δ and *myo2*-E1-Sup2 *myp2*Δ cells, respectively. (B) Time-lapse series of wild-type (*n*=31), *myo2*-E1 (*n*=18), *myo2*-E1-Sup2 (*n*=66), *myp2*Δ (*n*=36), *myo2*-E1 *myp2*Δ (*n*=15) and *myo2*-E1-Sup2 *myp2*Δ (*n*=56) cells expressing 3GFP-tagged myosin regulatory light chain (Rlc1-3GFP) as a contractile ring marker and mCherry-tagged tubulin (atb2-mCherry) as a cell cycle stage marker. Cells were grown at 24°C and shifted to 36°C for 3-4 h before imaging at 36°C (*t*=0 indicates the time Rlc1-3GFP nodes localize to the cell middle). Images shown are maximum intensity projections of *z*-stacks. Scale bars: 3 µm. (C) Quantification of timing of contractile ring assembly, dwelling and contraction in strains shown in B. Error bars represent s.d. (D) Kymographs from wild-type, *myo2*-E1-Sup2, *myp2*Δ and *myo2*-E1-Sup2 *myp2*Δ cells. Scale bars: 1 µm (horizontal axis) and 20 min (vertical axis). (E) Contraction rate determined from a graph of ring circumference versus time. Contraction rates of wild-type (*n*=33), *myo2*-E1-Sup2 (*n*=33), *myp2*Δ (*n*=21) and *myo2*-E1-Sup2 *myp2*Δ (*n*=39) cells show in B. Statistical significance was calculated by Student's *t*-test. Error bars represent s.d.

Having identified *myo2*-E1-Sup2, which reversed the defective growth and colony formation of *myo2*-E1, *myo2*-E1 *clp1*Δ and *myo2*-E1 *myp2*Δ, we used time-lapse imaging to quantitatively assess the dynamics of cytokinesis steps. We first studied the dynamics of actomyosin ring assembly and contraction in *myo2*-E1-Sup2 and *myo2*-E1-Sup2 *myp2*Δ cells. As controls, we studied these aspects of cytokinesis in wild-type, *myo2*-E1, *myp2*Δ and *myo2*-E1 *myp2*Δ cells. *myo2*-E1-Sup2 and *myo2*-E1-Sup2 *myp2*Δ cells resembled wild-type cells in morphology (Fig. 2A). We then imaged the dynamics of actomyosin ring assembly and contraction in wild-type, *myo2*-E1, *myo2*-E1-Sup2, *myp2*Δ, *myo2*-E1 *myp2*Δ and *myo2*-E1-Sup2 *myp2*Δ cells at 36°C. Actomyosin ring assembly was completed in ∼11-13 min in wild-type, *myo2*-E1-Sup2, *myp2*Δ, and *myo2*-E1-Sup2 *myp2*Δ (Fig. 2B,C and Movies 1 and 2). By contrast, improper actomyosin ring assembly required >33 min in *myo2*-E1 and *myo2*-E1 *myp2*Δ (Fig. 2B,C and Movies 1 and 2). These observations established that the ring assembly defect of *myo2*-E1 was fully reversed in *myo2*-E1-Sup2 and that Myp2p did not play an ancillary role in promoting actomyosin ring assembly in *myo2*-E1-Sup2.

When actomyosin ring contraction timings and rates were considered, we found that wild-type and *myo2*-E1-Sup2 completed the process of contraction in 20-22 min, suggesting that Myo2-E1-Sup2p was nearly as active as Myo2p in promoting actomyosin ring contraction (Fig. 2B,C,D and Movies 1 and 2). This view was also confirmed upon analysis of actomyosin ring contraction rates, which were comparable at ∼0.6 µm/min (Fig. 2E). As expected, ring contraction rates were slightly reduced in *myp2*Δ and *myo2*-E1-Sup2 *myp2*Δ, due to the ancillary role played by Myp2p in ring contraction (Bezanilla et al., 1997; Palani et al., 2017), and ring contraction was severely compromised in *myo2*-E1 and *myo2*-E1 *myp2*Δ cells (Palani et al., 2017). Thus, *myo2*-E1-Sup2 reverses the defective actomyosin ring assembly and contraction observed in *myo2*-E1.

We next created a strain bearing *myo2*-Sup2 mutation alone (i.e. only Y297C, without the G345R mutation) to investigate its phenotype (Fig. S1). We found that *myo2*-Sup2 was slightly slower in growth (Fig. S1A) and, although actomyosin ring assembly timing was comparable to that of wild-type cells, ring contraction was marginally slower (Fig. S1B-D). These observations suggest that G345R (*myo2*-E1) and Y297C (*myo2*-Sup2) are themselves significantly and partially compromised, respectively, but that they reciprocally suppress each other. Additional work will be required to understand the molecular basis of the mild ring contraction defect in *myo2*-Sup2.

**Fig. 3.**
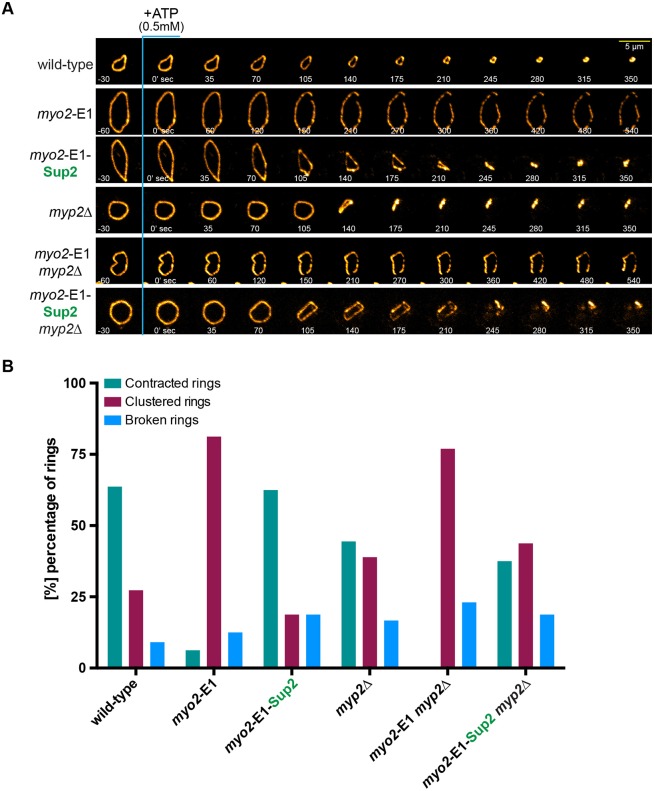
**Actomyosin rings from *myo2*-E1-Sup2 and *myo2*-E1-Sup2 *myp2*Δ cell ghosts undergo ATP-dependent contraction.** (A) Cell ghosts were prepared from wild-type (*n*=11), *myo2*-E1 (*n*=16), *myo2*-E1-Sup2 (*n*=16), *myp2*Δ (*n*=18), *myo2*-E1 *myp2*Δ (*n*=13) and *myo2*-E1-Sup2 *myp2*Δ (*n*=16) grown at 24°C. Ring contraction experiments were performed at 24°C and contraction activated by addition of 0.5 mM ATP. Images shown are maximum intensity projections of *z*-stacks. Scale bars: 5 µm. (B) Percentage of contracted, clustered and broken rings in strains illustrated in A.

Actomyosin ring contraction in *S. pombe* occurs concomitant with division septum assembly (Proctor et al., 2012; Zhou et al., 2015). Thus, it is possible that the ring contraction occurring in *myo2*-E1-Sup2 was due to ‘pushing’ by cell wall growth, as has been proposed (Proctor et al., 2012). To determine if ATP-dependent contraction was restored in *myo2*-E1-Sup2, we tested the ability of isolated actomyosin rings in cell ghosts prepared from wild-type, *myo2*-E1, *myo2*-E1-Sup2, *myp2*Δ, *myo2*-E1 *myp2*Δ, and *myo2*-E1-Sup2 *myp2*Δ to undergo ATP-dependent contraction. As shown before, rings prepared from *myo2*-E1 and *myo2*-E1 *myp2*Δ cells did not undergo ATP-dependent contraction (Mishra et al., 2013; Palani et al., 2017). Instead, these rings failed to contract and were either broken or became organized into clusters (Fig. 3A,B). By contrast, actomyosin rings isolated from wild-type, *myp2*Δ, *myo2*-E1-Sup2 and *myo2*-E1-Sup2 *myp2*Δ underwent proper contraction in the majority of cases (Fig. 3A,B). The efficiency of contraction in *myo2*-E1-Sup2 was comparable to that in wild-type cells (Fig. 3A,B). Similarly, the efficiency of contraction in *myo2*-E1-Sup2 *myp2*Δ was similar to that in *myp2*Δ cells (Fig. 3A,B). These experiments led us to conclude that the Y297C amino acid substitution fully restores function of Myo2-E1p to wild-type levels to accomplish actomyosin ring assembly and ATP-dependent ring contraction.

**Fig. 4.**
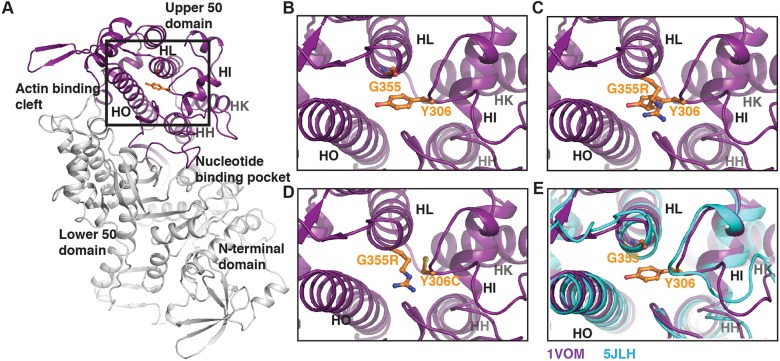
**Structural basis of suppression by *myo2*-E1-Sup2 mutant.** (A) Myosin motor domain highlighting the upper 50 kDa subdomain that contains the mutations G345R and Y297C, corresponding to G355 and Y306, respectively, in *Dictyostelium* myosin (PDB 1VOM shown in the figure). The insets show the zoomed view of the region. (B) G355 and Y306 in the wild type. (C) The steric clash introduced by G355R mutation modelled using PyMOL. (D) Probable removal of the steric clash by the double mutant G355R and Y306C. (E) Comparison with the rigor state conformation of myosin (PDB 5JLH, cyan) shows relative movement between helices in the region, highlighting the plasticity of the upper 50 kDa domain that may allow for a functional mutant at permissive temperatures.

What is the molecular basis of the defect in *myo2*-E1 and how is this reversed in *myo2*-E1 Sup2? The product of *myo2*-E1 is known to be stable at the restrictive temperature of 36°C (Mishra et al., 2005; Naqvi et al., 1999; Wong et al., 2000). Structural analysis of the upper 50 kDa sub-domain in the myosin head shows that the G345R mutation of *myo2*-E1 occurs at the C-terminal end of HL helix (corresponding to G355 in Fig. 4A,B). Mutation of the glycine to arginine results in the long side chain of arginine projecting into the pocket formed by HL, HO helices and Y306 (corresponding to Y297 of *myo2*-E1-Sup2 strain). This results in a steric clash between arginine and tyrosine, potentially resulting in instability of the myosin head domain during conformational changes accompanying the actomyosin contraction cycle (Stark et al., 2013). The strain caused by the steric hindrance is relieved by mutation of the bulky aromatic side chain of tyrosine to a smaller cysteine residue (Fig. 4D). This observation from the structure supports our experimental evidence of suppressor activity of the *myo2*-E1-Sup2 strain. Comparison with the actin-bound rigor state of the myosin head domain (5JLH; von der Ecken et al., 2016) shows that the helices HO, HL and loop containing Y306 undergo relative movement through various conformational states of the actomyosin cycle. We hypothesize that this relative flexibility of the helices within the upper 50 kDa domain potentially accommodates the G345R mutation at 24°C, and provides an explanation for the growth of *myo2-E1* strain at the permissive temperature, while affecting its growth in the higher temperature of 36°C.

In summary, through the isolation and characterization of *myo2*-E1-Sup2, we provide a molecular basis for the nature of the cytokinesis defect in *myo2*-E1 and the mechanism by which this defect is suppressed in *myo2*-E1-Sup2. The work also highlights the utility of a combined approach, involving classical genetics, imaging, reconstitution and structural analysis in understanding myosin II function. Such an approach with additional myosin II mutant alleles (e.g. *myo2*-S1 and *myo2*-S2; Wong et al., 2000) will provide important clues into the structure and function of myosin II in cytokinesis. Myosin II heavy chains are frequently mutated in cardiomyopathies (Huang and Szczesna-Cordary, 2015; Seebohm et al., 2009). In future, it will be interesting to introduce the equivalent of myosin II heavy chain mutations causing cardiomyopathies into fission yeast Myo2p, which should facilitate genetic suppressor screening as well as drug screens.

## MATERIALS AND METHODS

### Yeast genetics and culture methods

Log-phase cells were grown and cultured in yeast extract medium (YES) at permissive temperature (24°C). For the *in vitro* ghost experiment, cells were grown in Edinburgh minimal medium (EMM) with appropriate supplements, as described (Moreno et al., 1991). Temperature-sensitive mutants were shifted to 36°C for 3-4 h prior to fixation and imaging. Yeast stains used are detailed in Table S1.

### Screen for intragenic suppressors of *myo2*-E1

*myo2*-E1 *clp1*Δ cells plated on YES agar plates (∼2000 cells/plate) were exposed to UV (9000 µJ/cm^2^) for 6 s in a UV cross linker (UVP CL-1000 UV cross linker) as described previously (Palani et al., 2017). Colony PCR was performed from the colonies grown at 36°C using *myo2* internal primers.

### PFA fixation and fluorescence microscopy

Mid log-phase cells were grown at 24°C in YES and shifted to 36°C for 4-6 h before fixation. For visualization of DAPI and Anillin Blue staining, cells were fixed with 4% paraformaldehyde (PFA) and permeabilized with 1% Triton X-100 at room temperature for 10 min. Cells were washed three times with 1× PBS without detergent and stained with DAPI to visualize DNA and Anillin Blue to visualize septa and cell walls. Still images were acquired using a spinning disk confocal microscope (Andor Revolution XD imaging system, specifications are described below in the live cell imaging section). Imaging software Fiji was used to process the images.

### Live-cell imaging

For time-lapse live-cell imaging, log-phase cells were grown at 24°C and shifted to 36°C for 3-4 h prior to imaging. Time-lapse movies were acquired at 36°C in an incubation chamber for 3-4 h. CellAsic microfluidic yeast (Y04C) plates were used for time-lapse imaging. Time-lapse series were acquired using spinning-disk confocal microscope (Andor Revolution XD imaging system, equipped with a 100× oil-immersion 1.45 NA Nikon Plan Apo lambda objective lens, confocal unit Yokogawa CSU-X1, detector Andor iXon Ultra EMCCD and Andor iQ software). Fifteen *z*-stacks of 0.5 µm thickness were taken for Rlc1-3GFP and mCh-atb2 at 1 min intervals. Imaging software ImageJ or Fiji was used to process the images. Statistical significance was determined using Student's *t*-test. Prism 6.0 (GraphPad) software was used for quantification (s.d. and statistical significance).

For quantification of actomyosin ring contraction velocity, maximum intensity projections along the z-axis were generated from raw time-lapse data (7-µm-thick stacks sampled each 0.5 µm making 15 *z*-planes in total; *z*-stacks were taken at 1 min intervals). Next, assembled rings were outlined with line ROIs and kymographs of ring constriction were further generated for each ring. Finally, velocity of ring contraction was measured as inclination of a slope formed by the contractile ring edge in resulting kymographs. On average, 30∼50 rings were quantified per group.

### Cell ghost preparation and ATP-dependent *in vitro* ring contraction

Mid log-phase cells were grown at 24°C on minimal medium and rings were isolated as described (Huang et al., 2016; Mishra et al., 2013). Cell ghosts were prepared from wild-type, *myo2*-E1, *myp2*Δ, *myo2*-E1 *myp2*Δ and *myo2*-E1-Sup2 with or without *myp2*Δ cells. Rlc1-3GFP was used as a ring marker and images shown are maximum projections of *z*-stacks. Thirty *z*-stacks of 0.5 µm thicknesses were taken for Rlc1-3GFP. Experiments were done at 24°C. Cell ghosts from the wild type and mutants were treated with 0.5 mm ATP (*t*=0) and images were acquired at 30-45 s intervals for 20 min. Images were processed using Andor iQ and Fiji imaging software.

### Structure analysis and illustration

The myosin structures were downloaded from the Protein Data Bank (PDB ID, 1VOM, 5JLH; Smith and Rayment, 1996; von der Ecken et al., 2016). Structural analysis and illustrations were carried out using PyMOL (Schrodinger). Mutations of the relevant amino acids were carried out in PyMOL, and one of the rotamers chosen for the illustrations. Structural superpositions were performed for observing relative domain and helix movements.

## Supplementary Material

Supplementary information
