# Supplementary material for: Steric hindrance in the upper 50 kDa domain of the motor Myo2p leads to cytokinesis defects in fission yeast
Source: J Cell Sci. 2018 Jan 1;131(1):jcs205625. doi: 10.1242/jcs.205625 (PMC5818058; doi:10.1242/jcs.205625)
Supplement: Supplementary information [file joces-131-205625-s1.pdf]

Supplementary information

Figure S1

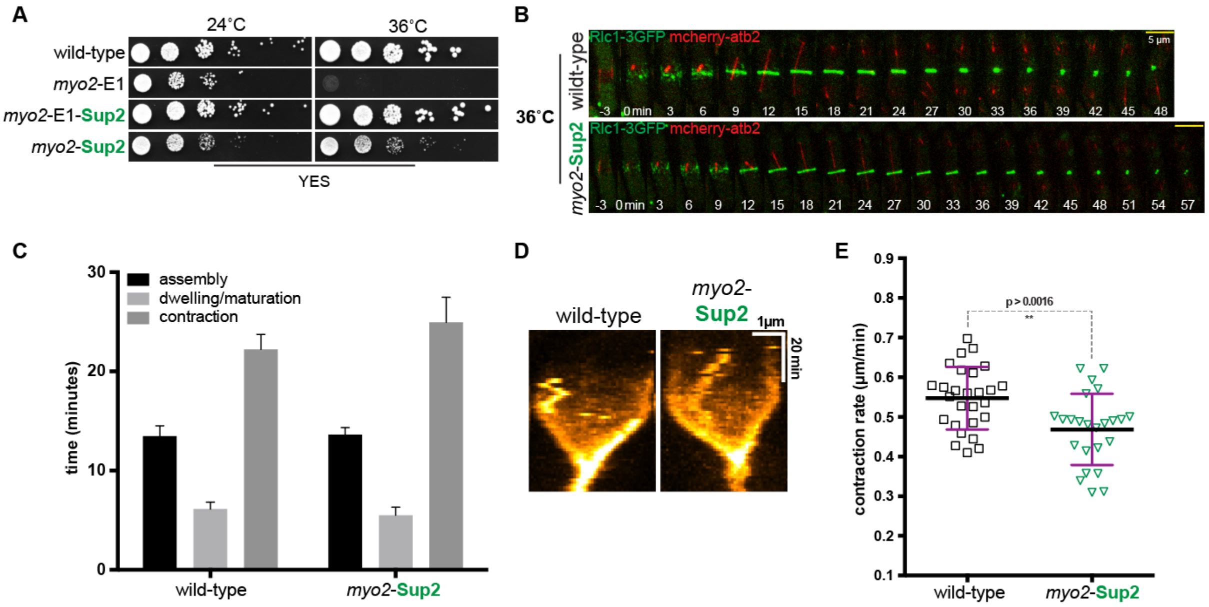

Figure S1. Characterization of *myo2*-Sup2 $^{(Y297C)}$ allele during cytokinesis.

A. Serial dilutions of wild-type, *myo2*-E1, *myo2*-E1-Sup2, *myo2*-Sup2 were spotted onto YES plates and grown for 4 days at 24°C and 36°C.

B. Mid-log phase cells were grown at 24°C and shifted to 36°C for 3-4 hrs before imaging at 36°C using cellAsic microfluidic chamber method. Time-lapse movie series of wild-type (n=23) and *myo2*-Sup2 (n=16) cells expressing 3GFP-tagged myosin regulatory light chain (*rlc1*-3GFP) as a contractile ring marker and mCherry-tagged tubulin (mCherry-*atb2*) as a cell cycle stage marker. Images shown are maximum intensity projections of Z-stacks. Scale bars represent 3 µm.

C. Timing of contractile ring assembly, dwelling/maturation and ring contraction. Quantification of Figure S1B. Error bars represent s.d.

D. Kymographs from wild-type and *myo2*-Sup2 cells. Scale bars represent 1 µm (horizontal axis) and 20 min (vertical axis).

E. Contraction rates of wild-type (n=27) and *myo2*-Sup2 (n=24) cells was quantified as described in Figure 2E. Statistical significance was calculated by student's t-test. Error bars represent s.d.

Journal of Cell Science • Supplementary information

Supplementary movies

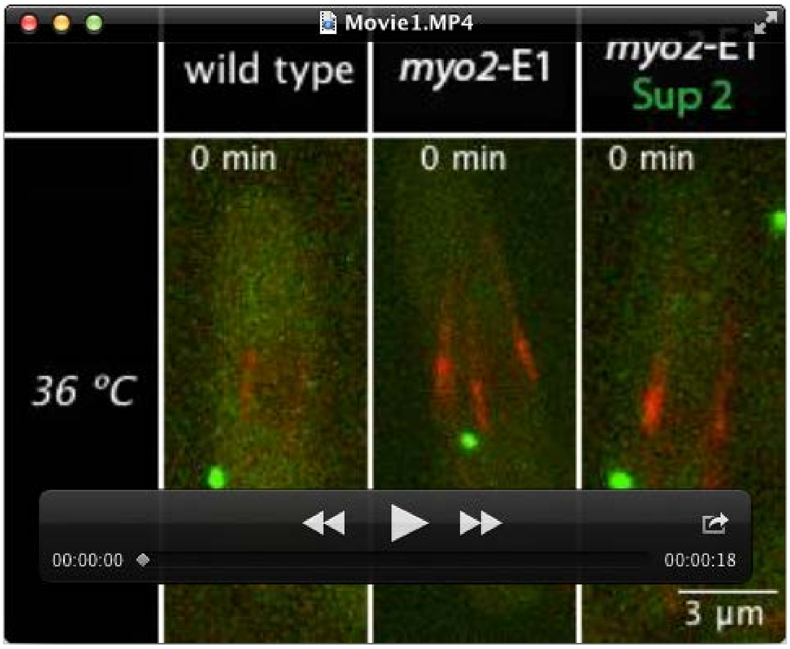

Movie 1. ***myo2*****-E1-Sup2 allele completely restored the defects of actomyosin ring assembly and contraction of *myo2*-E1.**

Representative time-lapse movies of mid-log phase cells of 3 genotypes (wild-type, *myo2*-E1, *myo2*-E1-Sup2) respectively. Images were acquired by spinning disk microscopy (Andor Revolution XD imaging system) at non-permissive temperature (36˚C). Cells were imaged using CellAsic microfluidic plate imaging method. Rlc1-3GFP (myosin regulatory light chain 1), which served as contractile ring marker and Alpha tubulin2 (mCherry-atb2) served as a cell cycle marker. Scale bar 3µm.

Journal of Cell Science • Supplementary information

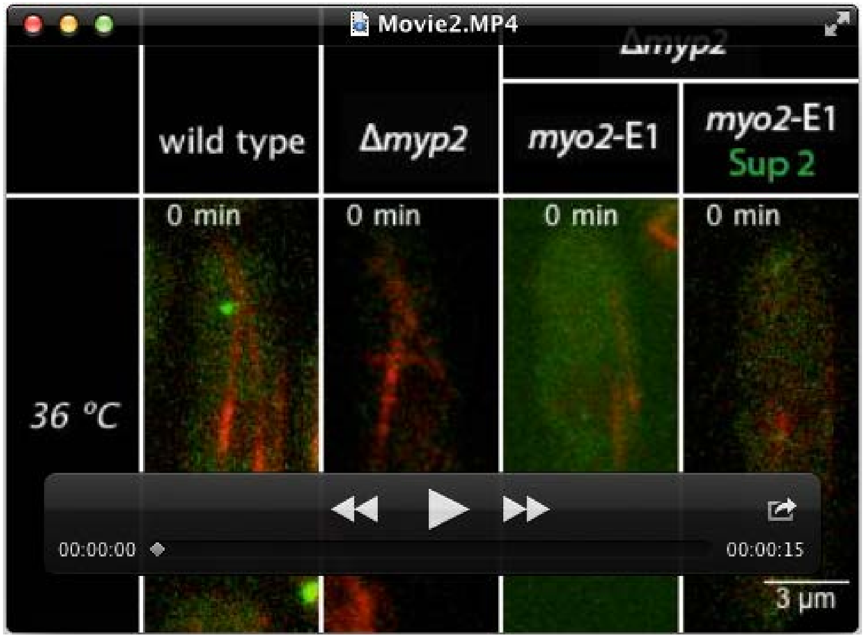

Movie 2. ***myo2*-E1-Sup2 did not require the presence of non-essential type II myosin (Myp2) to complete cytokinesis at the non-permissive temperature.**

Time-lapse movies of mid-log phase cells of 4 genotypes (wild-type, Δ*myp2*, *myo2*-E1 Δ*myp2*, *myo2*-E1-Sup2 Δ*myp2*) respectively. Images were acquired as described in Movie 1 and methods section. Scale bar 3µm.

Journal of Cell Science • Supplementary information

Table S1: List of strains used in this study

MBY192	*ura4*-D18, *leu1*-32, h-	Lab stock
MBY8841	mCherry-*atb2*::hph; *rlc1*-3GFP::kanMx6; *ura4*-D18 *leu1*-32 *ade6*-210 h+	(Palani et al., 2017)
MBY977	*clp1*::Ura4+, *ura4*-D18 *leu1*-32 *ade6*-21X h+	(Trautmann et al., 2001)
MBY151	*myo2*-E1 *ura4*-D18 *leu1*-32 *his3*-d *ade6*-21x h-	(Balasubramanian et al., 1998)
MBY2117	*myo2*-E1 *clp1*::*ura4*+ *leu1*-32 *ura4*-D18 *ade6*-21X	(Mishra et al., 2004)
MBY10024	*myo2*-E1[G345R] mCherry-*atb2*::hph; *rlc1-3GFP*::*kanMx6*; *ura4* -D18 *leu1*-32 *ade6*-210	(Zambon et al., 2017)
MBY8921	*myo2*-E1[G345R]-Sup2 [Y297C] mCherry-*atb2*::hph; *rlc1-3GFP*::*kanMx6*; *ura4* -D18 *leu1*-32 *ade6*-210	This study
MBY10075	*myp2*::natMX6 mCherry-*atb2::hph*; *rlc1-3GFP::KanMX6* *ura4*-D18 *leu1*-32 *ade6*-21X	(Palani et al., 2017)
MBY10097	*myp2*::natMX6 *myo2*-E1 mCherry-*atb2*::hph, rlc1-3GFP::KanMX6 *ura4*-D18 *leu1*-32 *ade6*-21X	(Zambon et al., 2017)
MBY10078	*myp2*::natMX6 *myo2*-E1[G345R]-Sup2 [Y297C] mCherry-*atb2*::hph; *rlc1*-3GFP::KanMX6 *ura4*-D18 *leu1*-32 *ade6*-21X	This study
MBY11624	*myo2*-Sup2 [Y297C] mCherry-*atb2*::hph; *rlc1*-3GFP::KanMX6 *ura4*-D18 *leu1*-32 *ade6*-21X	This study

Journal of Cell Science • Supplementary information

References:

Balasubramanian, M. K., McCollum, D., Chang, L., Wong, K. C., Naqvi, N. I., He, X., Sazer, S. and Gould, K. L. (1998). Isolation and characterization of new fission yeast cytokinesis mutants. Genetics **149**, 1265-75.

Mishra, M., Karagiannis, J., Trautmann, S., Wang, H., McCollum, D. and Balasubramanian, M. K. (2004). The Clp1p/Flp1p phosphatase ensures completion of cytokinesis in response to minor perturbation of the cell division machinery in Schizosaccharomyces pombe. J Cell Sci **117**, 3897-910.

Palani, S., Chew, T. G., Ramanujam, S., Kamnev, A., Harne, S., Chapa, Y. L. B., Hogg, R., Sevugan, M., Mishra, M., Gayathri, P. et al. (2017). Motor Activity Dependent and Independent Functions of Myosin II Contribute to Actomyosin Ring Assembly and Contraction in Schizosaccharomyces pombe. Curr Biol **27**, 751-757.

Trautmann, S., Wolfe, B. A., Jorgensen, P., Tyers, M., Gould, K. L. and McCollum, D. (2001). Fission yeast Clp1p phosphatase regulates G2/M transition and coordination of cytokinesis with cell cycle progression. Curr Biol **11**, 931-40.

Zambon, P., Palani, S., Kamnev, A. and Balasubramanian, M. K. (2017). Myo2p is the major motor involved in actomyosin ring contraction in fission yeast. Curr Biol **27**, R99-R100.

Journal of Cell Science • Supplementary information
